# A Review on Automatic Mammographic Density and Parenchymal Segmentation

**DOI:** 10.1155/2015/276217

**Published:** 2015-06-11

**Authors:** Wenda He, Arne Juette, Erika R. E. Denton, Arnau Oliver, Robert Martí, Reyer Zwiggelaar

**Affiliations:** ^1^Department of Computer Science, Aberystwyth University, Aberystwyth SY23 3DB, UK; ^2^Department of Radiology, Norfolk & Norwich University Hospital, Norwich NR4 7UY, UK; ^3^Department of Architecture and Computer Technology, University of Girona, 17071 Girona, Spain

## Abstract

Breast cancer is the most frequently diagnosed cancer in women. However, the exact cause(s) of breast cancer still remains unknown. Early detection, precise identification of women at risk, and application of appropriate disease prevention measures are by far the most effective way to tackle breast cancer. There are more than 70 common genetic susceptibility factors included in the current non-image-based risk prediction models (e.g., the Gail and the Tyrer-Cuzick models). Image-based risk factors, such as mammographic densities and parenchymal patterns, have been established as biomarkers but have not been fully incorporated in the risk prediction models used for risk stratification in screening and/or measuring responsiveness to preventive approaches. Within computer aided mammography, automatic mammographic tissue segmentation methods have been developed for estimation of breast tissue composition to facilitate mammographic risk assessment. This paper presents a comprehensive review of automatic mammographic tissue segmentation methodologies developed over the past two decades and the evidence for risk assessment/density classification using segmentation. The aim of this review is to analyse how engineering advances have progressed and the impact automatic mammographic tissue segmentation has in a clinical environment, as well as to understand the current research gaps with respect to the incorporation of image-based risk factors in non-image-based risk prediction models.

## 1. Introduction

Breast cancer is the most common cause of death in female cancer sufferers in both developed and developing countries [1]. The exact cause(s) of the majority of breast cancers still remains unknown [2]. By far the most effective way to tackle the disease is through early detection, precise identification of women at risk, and applying preventative measures. Within screening mammography, both mammographic density and parenchymal pattern have been established as image-based risk factors [2]. Current studies suggest that mammographic density is a major risk factor, even though it adds only little to the Gail model [3], and it is still not clear how to incorporate the factors as biomarkers into the risk prediction models [4].

Substantial advances have been made with computer aided mammography in breast cancer research and treatment. Mammographic risk can be assessed in a clinical environment based on subjective appraisal of mammograms using protocols such as BI-RADS (American College of Radiology's Breast Imaging Reporting and Data System) [5], which can lead to inter- and intraobserver variability [6]. Within computer aided mammography, the idea of developing a fully automatic and repeatable breast tissue segmentation using computer vision and machine learning techniques is to facilitate cancer risk classification. The major challenge is to segment a given mammogram according to density and/or parenchymal patterns so that the distribution and characteristic mixture of the breast tissue can be determined, leading to an accurate and objective breast cancer risk estimation. Many studies have been conducted in an attempt to achieve this; however there are still significant gaps in translating the newly acquired knowledge into clinical improvements [2]. In the recent publication, Harvey et al. [7] evaluated mammograms for approximately 3400 women. Breast density was calculated using automated software and used as an additional image-based risk factor for cancer risk prediction. Results indicated that reading of individual risk can be more accurate by incorporating image-based risk factors into established non-image-based risk model (e.g., the Gail [8] and the Tyrer-Cuzick [9] models). The initial results, based on a limited dataset, are promising; however, more investigations and evidence are required in order to fully establish and incorporate density into risk models in a clinical environment and to establish their place in changing clinical practice. A recent case-control study [10] compared six established digital mammographic density assessment methods and their ability to predict breast cancer risk. The selected approaches included three area based approaches and three fully automated volumetric methods. Area based approaches are referred to as mammographic image analysis over 2D breast tissue projection, whilst volumetric methods focus on assessment of the true physical volume of breast composition. With respect to patient care and economic sustainability, it is critical to have an effective and cost-effective analysis to evaluate the potential for personalised screening and prevention programmes and reduce anxiety and stress to the patient resulting from overscreening/treatment [11].

This paper presents a comprehensive review on automatic mammographic breast density and parenchymal segmentation methodologies developed over the past two decades, from its infancy in the early 90s to date. Note that mammographic breast density and parenchymal segmentation is referred to as mammographic tissue segmentation in the rest of the paper. The aim of this review is to analyse how engineering advances have progressed over these two decades and the impact automatic mammographic tissue segmentation has had in the clinical environment. Automatic mammographic tissue segmentation and measuring breast tissue composition for risk stratification (to adapt screening interval to risk) may be of prognostic value in distinguishing women with certain mammographic appearance likely to develop breast cancer, leading to a successful prevention and/or treatment. It should be noted that the focus of this review is to discuss how automated (as opposite to manual and semiautomated) segmentation of breast density and parenchyma is able to assist in the prediction of risk of developing cancer. These techniques do not aim to assess the risk of increased density masking breast cancer [12] when present although the two issues are clearly related. Note that the review does not cover aspects of abnormality detection or abnormality segmentation.

The remainder of the paper is organised as follows: Section 2 describes the established breast parenchymal patterns as seen in breast images and their interconnected relationships between different schemes, Section 3 briefly discusses mammograms and variations, Section 4 critically reviews the existing automatic mammographic tissue segmentation strategies in the literature, Section 5 provides discussion with respect to the development of automatic mammographic tissue segmentation, its progress, current state, and future beyond translational research in clinical practices, and Section 6 concludes the review.

## 2. Breast Parenchymal Patterns and Density Categories

Within screening mammography, a number of (mammographic risk assessment) schemes have been developed to estimate the likelihood of women developing breast cancer: Wolfe (1976) [13, 14], Tabár (1982) [15, 16], Boyd (1995) [17, 18], and BI-RADS (1993) [5, 19].

### 2.1. Wolfe

Wolfe [13] empirically categorised mammograms into four parenchymal patterns:

1. N1 (primarily fatty): mammogram is composed of fat and a few fibrous tissue strands.
2. P1 (≤25% prominent ducts): mammogram shows a prominent duct pattern and a beaded appearance can be found either in the subareolar or the upper axillary quadrant.
3. P2 (>25% prominent ducts): mammogram indicates severe involvement of a prominent duct pattern, which may occupy from one-half up to all of the volume of the parenchyma.
4. DY (dense fibrovascular tissue): mammographic features show an increase in density of the parenchyma, which may be homogeneous with a minor component of prominent ducts.

Wolfe's classification was found to be associated with breast cancer risk, and data analysis has revealed a progressive increase of as much as 37 times higher future cancer risk from N1 (the lowest risk) to DY (the highest risk) [14, 20]. Wolfe's pioneering study generated considerable controversy due to the (first) randomised controlled mammographic screening trial [21] and limited mammographic capability (e.g., breast positioning and compression) of the early 70s [14]. It should be noted that Wolfe's method cannot be repeated for its subjectivity of assessment. Wolfe's 1976 conclusions [13] have been criticised as erroneous and unrealistic, but the realisation of the association between parenchymal patterns and mammographic risk is indisputable, which contributed tremendously in research of image-based mammographic risk classification and leads the way for future clinical advances. The reader is referred to [14] for the key aspects of this study which caused debate of the controversies [13].

### 2.2. Tabár

Strongly influenced by Wolfe's work [21], Tabár and Dean [15] proposed a model based on a mixture of four mammographic building blocks composing the normal breast anatomy: nodular (N) densities mainly correspond to terminal ductal lobular units; linear (L) structures correspond to either ducts or fibrous or blood vessels; homogeneous (H) structureless densities correspond to fibrous tissues; radiolucent (R) areas are related to adipose fatty tissues. Mammograms were subdivided into five risk categories based on the distributions of the four building blocks (e.g., [N%, L%, H%, R%]) [16]:

1. T_I_ is composed of [25%, 15%, 35%, 25%], the lowest risk. Mammogram shows normal fibroglandular tissue with partial fatty replacement, where pathological changes can be easily perceived despite the fact that the breast may be “dense” radiologically.
2. T_II_ is composed of [2%, 14%, 2%, 82%]. Mammogram is characterised by the overrepresentation of radiolucent fatty tissue, which provides excellent background for radiologists to detect abnormalities.
3. T_III_ is similar in composition to T_II_, except that the retroareolar prominent ducts are often associated with periductal fibrosis. Neither of these patterns (i.e., T_II_ and T_III_) has nodular densities or diffuse fibrosis, and the overrepresentation of radiolucent fatty tissue makes pathological lesions relatively easier to detect through mammography.
4. T_IV_ is composed of [49%, 19%, 15%, 17%]. Mammogram is dominated by prominent nodular, linear densities and appears to be resistant to the process of involution, which makes perception of pathological lesions difficult on mammograms.
5. T_V_ is composed of [2%, 2%, 89%, 7%], the highest risk. Mammogram is dominated by extensive homogeneous structureless fibrous tissue, which limits the capabilities of mammography to demonstrate the normal anatomy and reveal small pathological lesions.

Tabár's approach is based on mammographic anatomic (pathologic) correlations rather than pattern reading alone (e.g., Wolfe classification), and the primary difference between Wolfe and Tabár's classification is Tabár's T_I_ [22, 23]. Tabár's definitions of mammographic risk patterns are more discriminatory than Wolfe's, helping to increase reproductive accuracy of the classification. The reader is referred to [23, 24] for a detailed comparison with respect to these two risk schemes.

### 2.3. Boyd

In a subsequent study to [13], Wolfe et al. [85] described a stronger association between mammographic density and breast cancer risk. This led Boyd et al. [17] to develop a method to measure mammographic percentage density using a computer aided technique, which marked movement away from describing patterns to objective assessment using tissue percentages. In particular, the risk categories are defined using a thresholding method [86], also known as the Cumulus interactive threshold software (University of Toronto), which is considered to be the “gold standard” tool for density measurement [81, 87]. With the Cumulus, the reader can identify the boundaries of the breast tissue, define the threshold for dense tissue on the mammogram, and measure the total area of the breast. The percentage of mammographic density is examined to estimate the proportion of fibroglandular tissue (as opposed to fat) of the breast and is divided into six class categories (SCC):

1. SCC_1_, density = 0%,
2. SCC_2_, density < 10%,
3. SCC_3_, 10% ≤ density < 25%,
4. SCC_4_, 25% ≤ density < 50%,
5. SCC_5_, 50% ≤ density < 75%,
6. SCC_6_, density ≥ 75%.

Subsequent follow-up studies [17, 88] have established a significant independent association between overall breast density and future breast cancer risk, which indicated a much lower magnitude (4-5 times increase) than the 37 times increase initially proposed by Wolfe [13].

### 2.4. BI-RADS

A series of studies (e.g., [85, 88, 89]) indicated that the percentage of dense breast tissue is highly associated with breast cancer risk. BI-RADS [5] was designed as a quality assurance tool to standardise mammography reporting, in order to reduce confusion in breast imaging interpretations and to facilitate outcome monitoring. Four breast compositions were identified as follows:

1. B_1_, the breast being almost entirely fat (<25% glandular);
2. B_2_ (25%–50% scattered fibroglandular densities);
3. B_3_ (50%–75% heterogeneously dense breast tissue);
4. B_4_, the breast being extremely dense (>75% glandular).

BI-RADS, widely used throughout North America and much of Europe [90], covers the significant relationship between increased breast density and decreased mammographic sensitivity in detecting cancer [14, 91].

Area based assessments can be subjective and imprecise. Volumetric density is a relatively new concept. The developed volumetric measuring techniques work out what kind of tissues must have been present by assessing breast composition at each pixel by calculating the X-ray attenuation between that pixel and the X-ray source [83]. Volumetric breast density and visual assessment are strongly correlated [10]; however, visual density categories as described in BI-RADS based on 2D projection cannot be directly used for volumetric breast density assessments. For example, visual density areas as shown in side on views of breasts under compression can be the same/similar due to superimposition; the measurements can be very different if measured based on volumetric breast density. The fifth edition of BI-RADS Atlas [92] included changes in breast density reporting categories with emphasis on volumetric assessments. The updated BI-RADS helps clarify what breast density assessment should be and distinguishes density from other BI-RADS assessments. The latest edition indicates that, in a clinical environment, focus of density as a risk factor has now moved towards density as a masking factor.

### 2.5. Correlations between Different Schemes

Muhimmah et al. [24] investigated the (Spearman's) correlations (*r*_*s*_) between the four different schemes (i.e., Wolfe, Boyd, BI-RADS, and Tabár) using the MIAS (Mammographic Image Analysis Society) database [93], which is a publicly available, digitised database. Results indicate risk classification mappings between the four schemes as follows.

- Wolfe → Boyd: N1 →SCC_1_, P1-2 → SCC_2–5_, and DY →SCC_6_; *r*_*s*_ = 0.93; this is in line with reported results (*r*_*s*_ = 0.81) by Brisson et al. [91];
- Wolfe → BI-RADS: N1 →B_1_, P1 →B_2_, P2 →B_3_, and DY →B_4_; *r*_*s*_ = 0.93.
- Wolfe → Tabár: N1 →T_II_, P1 →T_III_, P2 →T_IV_, and DY →T_V_; *r*_*s*_ = 0.93 excluding T_I_ due to the weak correlation with any of the Wolfe patterns [24]; this is in line with results in [22, 23];
- Boyd → BI-RADS: SCC_1_→B_1_, SCC_2-3_→B_2_, SCC_4-5_→B_3_, and SCC_6_→B_4_; *r*_*s*_ = 0.91;
- Boyd → Tabár: SCC_1_→T_II_, SCC_2-3_→T_III_, SCC_4-5_→T_IV_, and SCC_6_→T_V_; *r*_*s*_ = 0.93;
- BI-RADS → Tabár: B_1_→T_II_, B_2_→T_III_, B_3_→T_IV_, and B_4_→T_V_; *r*_*s*_ = 0.96 excluding T_I_ [24]; a recent study [94] indicated a strong direct correlation between these two schemes with T_I_ taken into account, where B_1_→T_II/III_, B_2_→T_I_, B_3_→T_IV_, and B_4_→T_V_; *r*_*s*_ = 0.92; note that Tabár's T_II_ and T_III_ have the same tissue composition; see Section 2.2.

**Figure 1:**
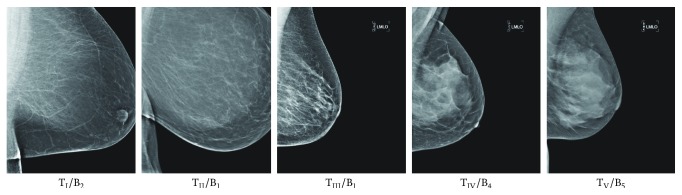
Example mammographic images with Tabár (T) risk classifications from low to high and their equivalent according to BI-RADS (B) scheme.

**Figure 2:**
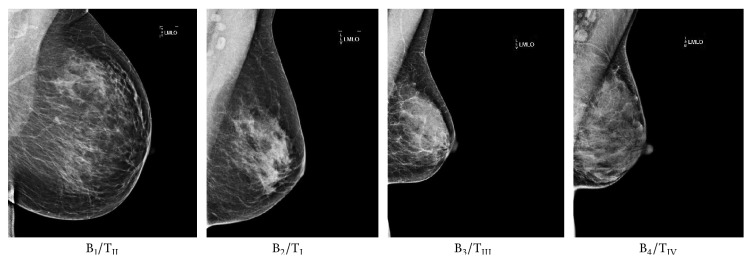
Example mammographic images with BI-RADS (B) density categories from low to high and their equivalent according to Tabár (T) risk classification.

Strong correlations were established between Wolfe, Boyd, and BI-RADS categories [24] but the correlations with Tabár categories are less straight forward due to Tabár's T_I_. According to the literature, these correlations have not been investigated using other mammographic modalities (e.g., digital mammography and tomosynthesis), with the exception of one study [94] which investigated the correlation between BI-RADS and Tabár categories using a private digital mammography database. Mammographic parenchymal pattern and percentage density (PD) have shown being strongly associated with breast cancer risk in the literature [95–97]. However, Brisson et al. [91] suggested that PD provides more information on breast cancer risk than Wolfe's parenchymal patterns. Once PD is taken into account (e.g., using BI-RADS scheme), parenchymal pattern information is redundant. Tabár's scheme seems to capture something more than just density assessment, which could be useful in temporal analysis of breast parenchymal changes. However, it is still unclear whether this additional information from Tabár's scheme is related to breast cancer risk [23]; therefore further investigations are warranted. Example mammographic images assessed based on Tabár and BI-RADS are shown in Figures 1 and 2, respectively.

## 3. Screen Mammography

Early detection through breast screening programmes is by far the most effective way to improve survival rate [98]. There are multiple breast imaging modalities; mammography, ultrasound, thermography, PET (positron emission tomography), MRI (magnetic resonance imaging), CT (computed tomography), scintimammography, optical imaging, and electrical impedance tomography (EIT) based imaging have all been used for different purposes. The clinical role for nonmammographic modalities is often to provide additional information when the results of mammograms are indeterminate or of limited utility [99]. This review focuses on evaluation of density using (X-ray related) mammographic modalities, and other modalities are outside the current scope. The reader is referred to [98] for a detailed overview and [100] for a comparison of breast imaging modalities, and the associated biomarkers used for early detection can be found in [101].

Mammography is the gold standard method in detection of early stage breast cancer before lesions become clinically palpable. The most criticised aspect of screening mammography is that, for women with dense breasts, the sensitivity of mammography is significantly reduced leading to concerns over unnecessary biopsies and treatment [102, 103]. This may lead to another harm to the patient (e.g., physical, emotional, financial, and/or psychological) [104]. Despite these limitations, mammography as an initial examination can have very good sensitivity and specificity when compared to other modalities (e.g., PET, CT, and MRI). However, the reading and assessment of mammogram densities can be highly subjective with only moderate agreement among radiologists [24, 105, 106]. Note that modalities such as ultrasound and MRI can have better sensitivity in some cases but with a corresponding loss of specificity. Clinically these modalities are most often used as an adjunct to mammography, whether 2D or 3D, and as follow-up examination to investigate specific mammographic findings. Mammography is a low cost simple procedure, which is affordable for all population screening [98]. Therefore, there is a huge incentive to develop computer aided mammographic analysis approaches, in order to deliver objective and accurate results in an effective and cost-effective way and to facilitate early detection and precise identification in a clinical environment.

### 3.1. Mammographic Image Quality Aspect

Mammographic image quality can be considered an indication of clarity with which radiologically significant details can be perceived in an image. Superb image quality is imperative for reliable detection and accurate characterisation of breast parenchyma and is expected to be beneficial for breast imaging analysis. Broadly speaking, there are two types of mammography including 2D mammographic projection (e.g., screen-film mammography (SFM) and full field digital mammography (FFDM)) and 3D digital breast tomosynthesis (DBT).

#### 3.1.1. SFM and FFDM

Clinical evaluation has indicated that SFM and FFDM are similar in their ability to detect cancer [107]; however, FFDM is more effective at finding cancer in certain groups of the population, such as women who are premenopausal or perimenopausal, under the age of 50, and have dense breasts. This indicates that in this subgroup some anatomical regions are better visualised by FFDM than SFM. In particular, FFDM demonstrated improved image quality with significantly better depiction of the nipple, skin, pectoral muscle, and especially contrast in parenchymal and fatty tissue [108]. Note that digital mammography imaging generates two types of images for analysis, raw (“for processing”) and vendor postprocessed (“for presentation”), of which postprocessed images are commonly used in clinical practice.

#### 3.1.2. DBT

Routine screening mammography relies upon a select number of views, for example, Craniocaudal (CC) and Mediolateral Oblique (MLO) view, to assess breast tissue structures [109]. With 2D projections, it can be difficult to separate normal glandular tissue from tumours. One of the biggest challenges to screening radiologists is to interpret superimposed fibroglandular tissue (anatomical noise) in the image, in which pathological structures can be obscured and remain undetected. In some cases this can mimic lesions leading to false positive results, unnecessary recalls for additional screening, and/or biopsy. DBT is a recent advanced image acquisition technology, in which the conventional mammography technique has been modified to acquire a 3D view of the breast; a series of thin-resolution images are aggregated to generate a 3D image of the breast. The reconstructed format eliminates image superimposition and allows images of submillimetre cross sections to be analysed, increasing the conspicuity of features that are often obscured by overlapping structure in a single-projection view [110]. A newly developed 2D imaging “modality” called C-View (synthetic 2D view) can be generated from the 3D DBT data during the mammography exam [111], eliminating the need for additional 2D exposures. Initial clinical studies have shown that screening with C-View imaging may result in clinical performance superior to that of a conventional 2D mammogram [112]. However, further evaluation and validation are needed to verify the usage of C-View in a clinical environment. Note that density assessment between area based 2D projection and 3D DBT can be very different. Clinical investigation is currently under way to establish the relationships between density assessment using these two very different modalities.

## 4. Automatic Breast Density and Parenchymal Segmentation

In principle, there are three approaches to mammographic tissue segmentation, manual, semiautomatic (interactive), and fully automatic. Although more effective than manual segmentation with respect to speed and efficiency, semiautomatic methods have several limitations; the interactive segmentation and labelling of mammograms can still be subjective and time consuming and require operator training. Such approaches make large studies and clinical usage costly. Defining parameters in a subjective manner could introduce observer bias. However, manual and semiautomatic approaches can sometimes be a good idea for obtaining “ground truth” to facilitate other studies. The focus of this review is on fully automatic approaches to mammographic tissue segmentation, with manual and semiautomatic approaches excluded.

**Figure 3:**
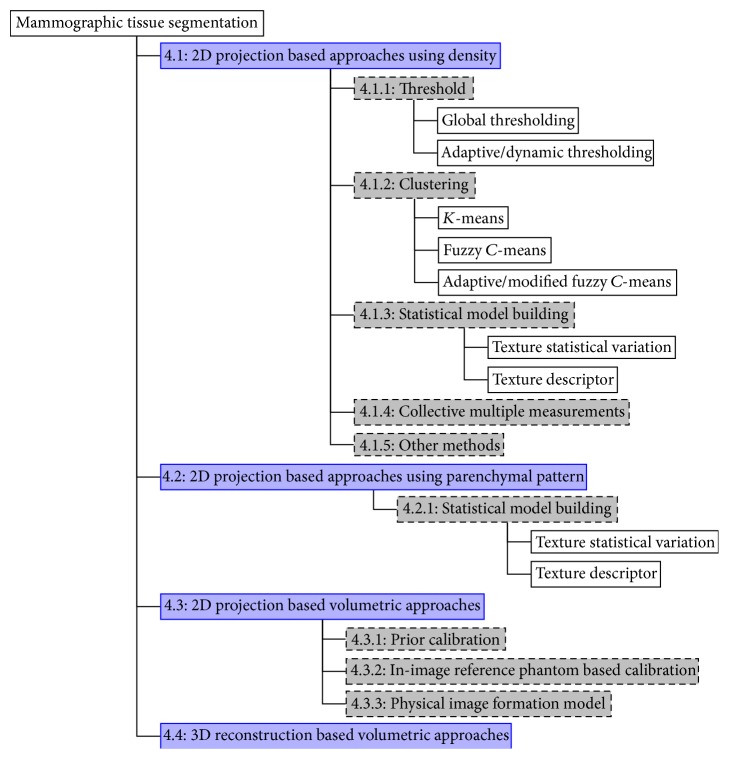
A tree representation for mammographic tissue segmentation categorisation based on combination (e.g., shaded in light purple) of technological advances and image modalities. Subcategorisation (e.g., shaded in light grey) is based on core segmentation principles.

A large quantity of literature was reviewed in order to illustrate the progress and advances in mammographic tissue segmentation; this section is separated into four categories according to a combination of two technological advances, imaging modalities (i.e., 2D projection and 3D reconstruction) and segmentation principles (i.e., based on 2D projection or volumetric data). It is ideal to use categorisation based on a combination of the technological advances over the past two decades, so that the separation within the literature is according to the timeline of the developments. The reader is referred to Figure 3 which shows a tree representation for mammographic tissue segmentation categorisation used in this review.

Mammographic tissue segmentation is often used as an intermediate stage prior to deriving certain features from the segmentation for risk classification. Some studies are closely related and in some cases the same methodologies evolve over time with improved results. In order to fully focus on breast density and parenchymal segmentation, other aspects such as comparisons of classification techniques used for the follow-up risk classification are briefly noted, as these are considered to be separate modules/steps in the process pipeline. However, risk classification results are sometimes used to indicate the correctness of the segmentation when the evaluation of the segmentation quality is not available. The reviewed mammographic tissue segmentation is explicitly developed for breast tissue separation within the breast region and does not cover the detection/segmentation of abnormalities (e.g., mass and microcalcification), not does it cover the separation of the breast area in mammograms. Note that, in the literature, breast tissue density and dense tissue are often referred to as parenchymal patterns, fibroglandular disk, and parenchymal density.

### 4.1. 2D Projection Based Approaches Using Density

**Figure 4:**
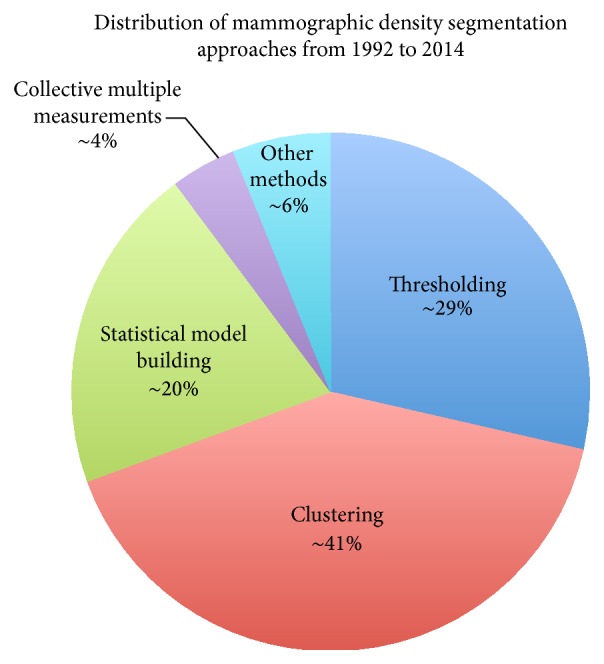
Distribution of mammographic density segmentation using 2D projection based approaches.

The majority of mammographic tissue segmentation uses digitised SFM or FFDM. Based on the core segmentation principles, they can be categorised into five groups: thresholding, clustering, statistical model building, collective multiple measurements, and other methods.  Figure 4 shows the distribution of the studies conducted with respect to the five groups.

#### 4.1.1. Thresholding

To overcome observer bias in choosing threshold values subjectively, various studies were conducted to explore techniques to determine a threshold value automatically in order to achieve consistent segmentation objectively in a high-throughput manner. High intensity dense and low intensity fatty breast tissue can be separated intuitively based on the mammographic pixels values. This led to the development of automatic threshold determination for mammographic tissue segmentation. Single threshold value approaches are difficult due to mammographic density inhomogeneity leading to inaccurate mammographic tissue segmentation and inaccurate breast density estimation. To improve this, adaptive/dynamic threshold based methods have emerged. A summary of representative studies using thresholding techniques can be found in Table 1. Note that automated thresholding is simple but it has not worked reliably to date across databases. Various studies indicated good results with the developed approaches versus risk; however, they may have been heavily trained on specific databases.

*Global Thresholding.* Early studies investigated various discriminant functions for breast density based segmentation, and the following studies are some examples. Matsubara et al. [25] applied a two-stage segmentation technique. Fatty and dense tissue were segmented using variance histogram analysis. The segmented dense tissue was then further grouped into either dense or semidense tissue by a binary technique and the threshold values were determined by discriminant analysis. Alternatively, Saha et al. [26] developed an automatic method to segment fatty and dense breast tissue using scale-based fuzzy connectivity methods [113]. Dense breast tissue was segmented as a set of fuzzy connected objects using automatically determined threshold values. The optimal threshold was derived by minimising a second-order statistic threshold energy function, computed by considering spatial arrangements of pixel intensities. Sivaramakrishna et al. [27] used a modified version of Kittler's optimal threshold procedure [114] for dense and fatty breast tissue segmentation, whilst Olsén and Mukhdoomi [28] used minimum cross-entropy [115] to obtain an optimum threshold for segmenting glandular tissue automatically. Early investigations assumed that regions of glandular tissue have significant differences in brightness compared to the surrounding fatty tissue. Many histogram-based techniques focused on detecting intensity peaks which may indicate dense tissue or abnormalities, regardless of glandular tissue intensity and texture variation. Such an approach is limited because fibrous tissue is often embedded in fatty tissue, and the appearance of glandular tissue can be ranging from bright, fluffy blobs, to sparse lines. It is an inherent disadvantage for 2D mammographic projections to have superimposed tissue and overlapping structures which cause variation in glandular/fatty intensity.

Ferrari et al. [31] presented a technique to segment the fibroglandular disc in mammograms based on the statistical modelling of breast density using a weighted Gaussian mixture model. The parameters of the model and the number of breast tissue classes were determined using the expectation-maximization (EM) algorithm. Ferrari et al. [32] improved the technique by segmenting the fibroglandular disc in mammograms using four weighted Gaussians. The different density functions of the model were represented by the mixture of Gaussians corresponding to specific density classes in the breast. The parameters of the model and the number of breast tissue classes were also determined using the EM algorithm, where the number of Gaussian kernels of the model was determined by an information theoretic approach (i.e., minimum description length method [116]). The shortcoming in identifying the fibroglandular disc in the related studies [31, 32] was the parameter initialisation in the EM algorithm which can lead to convergence to local maxima, causing poor segmentation results. Similar to Ferrari et al. [32], El-Zaart [33] used a statistical approach for detecting the fibroglandular disc. The key difference between the two approaches is that Ferrari used Gaussian mixture modelling while El-Zaart used Gamma mixture modelling. The EM technique with a Gamma distribution was developed to estimate the statistical histogram parameters. Evaluation indicated that Gamma based method detected the fibroglandular disc regions more precisely, while the Gaussian based method falsely detected more regions that are not part of the glandular discs. The mixture of Gaussian distributions was used to model various breast parenchyma and is ideal for symmetric data; however, experimental results showed that the image histograms are not always symmetric. For this reason, Gamma distributions are more appropriate for modelling symmetric and nonsymmetric histograms of mammographic images, from which the thresholding values were selected at the valleys.

Lu et al. [30] adapted the semiautomatic breast density segmentation proposed by Boyd et al. [17], and modified the algorithm for automatic measuring of breast density from raw FFDM. A multiple regression model analysis was developed utilising image acquisition parameters and pixel intensity statistics to derive threshold values for mammographic tissue segmentation. Such an approach can be affected by various factors that influence the signal intensity recorded by the digital detector, including the estimation error of the compressed breast height, the heel effect, quantum mottle (noise), beam hardening, and detector nonuniformity. A FFDM unit may exhibit temporal changes due to ageing of the X-ray tubes and variation between FFDM unit manufacturers; moreover differences between radiology facilities can also be an issue. The approach can be problematic as the parameters and parameter estimates of regression models may change from installation to installation, site to site, and analyst to analyst.

*Adaptive/Dynamic Thresholding.* Zhou et al. [34] investigated an approach in which the image was first classified by hand according to the characteristic features of the grey-level histogram into one of the BI-RADS density categories using a rule based classification. With the known BI-RADS density category and the shape of the histogram (e.g., unimodal, bimodal, or multimodal), the density segmentation was achieved by using combined discriminant analysis [117] and maximum entropy principle [118] based threshold selection methods. Successful segmentation strongly depended on a correctly classified mammogram, which would result in selecting the optimal threshold. The developed method was used in a clinical study [119], and results suggested that misclassified histograms occurred more often on extremely dense and fatty mammograms, but the overall breast density estimation was more accurate than the radiologists' visual estimation. However, because the configured parameters for threshold determination were dataset specific and the segmentation was performed after density classification, it was concluded in [119] that such an automatic approach cannot yet be used as a stand-alone density measurement tool.

Kim et al. [36] developed a scheme for breast density estimation using statistical (e.g., standard deviation) and boundary information (e.g., edge magnitude) to compute an optimal intensity threshold between dense and fatty tissue in order to define dense and nondense areas. One key hypothesis in the study is that boundaries between the dense and fatty portions of the breast usually have high values of gradient magnitude. Therefore an iterative search was used and the optimal threshold was determined by combining these features to best divide the fat and dense regions. Nickson et al. [37] adopted this method for mammographic density segmentation and noticed that often a false optimal threshold is produced, which separates a narrow band of low intensities along the breast skin line from the rest of the breast. Nickson et al. modified the method, named AutoDensity, to converge to an intensity level that separates dense from fatty tissues. This is achieved by performing an iterative search for the optimal threshold while decreasing a region of interest within the breast until a stopping condition is met. The modified method was used on a large population screening programme, and the automated measurement of breast density from digitised SFM using AutoDensity performs similarly (modest correlation) to the Cumulus (a semiautomated user-assisted PD estimation method) [17].

#### 4.1.2. Clustering

Clustering techniques have been widely used for mammographic density segmentation. The principle is to segment the breast tissue based on pixels with similar tissue appearance. Three groups can be identified, general (hard clustering), fuzzy (soft) clustering, and EM hierarchical clustering. A summary of representative studies using clustering techniques can be found in Table 2.

*General Clustering.* Oliver et al. [38] employed the *K*-means algorithm [120] for mammographic density segmentation, and breast density was categorised into fatty, glandular, and dense tissue. A set of morphological and cooccurrence matrix [121] based texture features were extracted from the segmented areas; these features were used for mammographic risk classification based on a leave-one- (image) out methodology. Results indicated that the risk classification accuracies decreased dramatically when including the glandular class; as dense breast area increases, more variations appear between the tissue clusters. This suggested that the *K*-means clustering based segmentation may be limited in dealing with inter- (density) class variation. Other texture and/or intensity based features can also be used in such a clustering based mammographic tissue segmentation; however, computational efficiency may decrease as the number of (combined) features increases. Dimensionality reduction can be used to remove redundant features. Strange et al. [39] used a manifold learning technique to preserve certain manifold properties using a large scale Kernel Principal Components Analysis (PCA) [122], as a means of reducing the data dimensionality prior to a clustering based mammographic tissue segmentation. *K*-means clustering was used to assign either fatty or dense tissue class to each image pixel based on the nearest cluster in low-dimensional manifold space. Results suggested that such a manifold learning was good for BI-RADS category 4 but less satisfactory for the other BI-RADS density categories.

**Figure 5:**
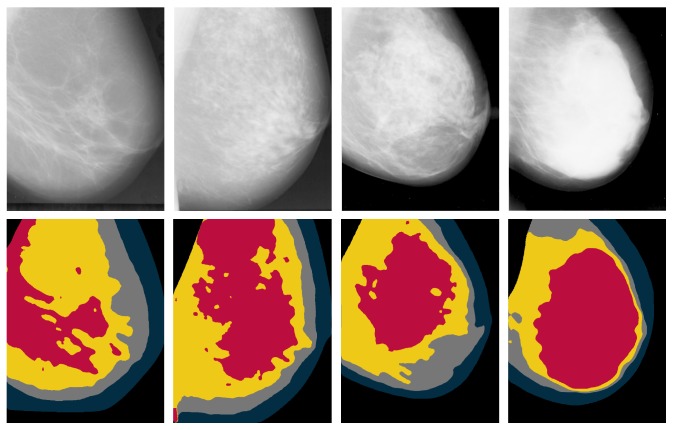
Example mammographic density segmentation. Images (digitised mammograms) from left to right are rated with BI-RADS 1–4, respectively. Fatty, semifatty (e.g., scattered fibroglandular tissue), semidense (e.g., heterogeneous dense tissue), and dense tissue are colour coded as navy, grey, yellow, and red, respectively.

*Fuzzy Clustering.* Oliver et al. [41, 42] used fuzzy *C*-means (FCM) [123] (an extension of *K*-means) to allow a cluster to be associated with one or several classes (i.e., fatty, glandular, and dense tissue); this is referred to as fuzzy membership function. In this improved approach, the classification was performed using the DDSM (Digital Database of Screening Mammographies) data [124], a publicly available digitised mammogram database. When compared with [38] the risk classification accuracies were improved. This may indicate that FCM produced more accurate segmentation, which resulted in more discriminative breast tissue features. Oliver et al. [43] quantitatively compared FCM with normalised cuts [125] and mean shift [126] for clustering based mammographic tissue segmentation. With respect to the subsequent risk classification, results indicated that FCM outperformed the other approaches. Some aspects regarding the actual density segmentation were lacking in related studies (e.g., [38, 41]) but were addressed in [45, 46]. In particular, the FCM approach to mammographic tissue segmentation was compared with other techniques. First, a fractal technique [127] was used to recursively split an image into quadrants, where the stopping criteria were determined based on local histogram measures on the consistency of uniform tissue. The quality of the fractal based approach resulted in pixelated segmentation due to its quad tree structure based splitting and analysis. Second, a statistical region based approach supported by the Fisherfaces algorithm [128] was used, in which a set of mammographic patches containing either dense or fatty tissue was used to model breast tissue. Subsequently, a model driven mammographic tissue segmentation was performed. Such an approach resembles rather large and coarse segmentation due to the block tissue analysis that could be less effective when tissue variations are small. Third, a multiple thresholding based on information theory (i.e., excess entropy [129]) was investigated. The excess entropy based algorithm loses accuracy in areas where the dense and fatty tissue boundary is less clear. The FCM outperformed the other approaches and produced more anatomically realistic segmentation, which led to satisfactory risk classifications. Tortajada et al. [47] extended and consolidated the segmentation framework in [46] for mammographic density segmentation using FFDM. A novel peripheral enhancement technique was developed to enhance texture appearance of uncompressed-fatty tissue near the breast skin line. Mammographic segmentation was significantly improved near the breast peripheral areas, leading to more correct features derived from the segmented dense tissue, and higher risk classification accuracies were achieved. Example mammographic segmentation can be found in Figure 5.

*Adaptive/Modified Fuzzy C-Means.* Chen and Zwiggelaar [48] proposed a modified FCM algorithm for mammographic density segmentation using the EPIC (European Prospective Investigation on Cancer) database [130]. The modified FCM incorporates local spatial and intensity information based on an adaptive local window filter; the weighting coefficients within the local window are used to differentiate the neighbouring pixels. The clustering performance on the intensity histogram of the filtered image is faster than conventional FCM. The conventional FCM algorithm uses grey-level information at a single pixel as the feature space and this contains no spatial contextual information, which makes it very sensitive to noise and intensity inhomogeneities. Segmentation results indicated the robustness of the modified FCM in dealing with intensity inhomogeneities with different density categories. Visual assessment indicated that local window filtering was able to eliminate intensity inhomogeneities and avoid excessive blur.

Keller et al. [49] presented a novel multiclass FCM algorithm for automated identification and quantification of breast density, which is optimised for the imaging characteristics of digital mammography. The proposed algorithm involves an adaptive histogram-based method to estimate the number of clusters, which uses the tissue properties of the specific mammogram followed by segmentation through clustering using linear-discriminant analysis (LDA). The classifier combines imaging and patient characteristics to achieve optimal segmentation through cluster merging. A set of FFDM was used in the evaluation, and a strong correlation was observed between the estimated PD and radiological “ground truth” using BI-RADS density categories. Results also indicated relatively poor performance when compared to identifying breast dense tissue based on two-class FCM paradigm using digitised SFM. This may be due to the fact that the majority of grey-level intensity profiles of breast tissue as extracted from FFDM tend to be multimodal. This finding is not in line with the results reported in [46], in which a good visual agreement was observed between FCM and expert annotations. This may be due to the fact that the clustered features are different; cooccurrence matrices and grey-level intensities features were used in [46] and [49], respectively. Keller et al. [50] extended the adaptive multicluster FCM approach to estimate breast PD in both raw and postprocessed FFDM images. This is expected to be beneficial in terms of direct clinical application and retrospective analysis.

*Expectation-Maximisation.* Aylward et al. [51] investigated a mixture modelling technique to differentiate fatty and dense breast tissue. The statistical modelling of the breast components was based on pixel intensity distribution sampling, using Gaussian mixture models, but no texture aspects were taken into account. The parameters of the mixture were iteratively determined using the EM algorithm, maximising the log-likelihood of the data representing the distribution [131]. To quantify accuracy, automatic dense tissue estimates were compared with the “ground truth” provided by experts. A set of images from three different mammography unit manufacturers was used in the evaluation. Whilst breast density estimates were satisfactory, in some cases, the separation of fat and dense tissue was not achieved. Nevertheless, results from this early study showed possible intensity distribution discrepancy (e.g., variation in breast tissue contrast) when using images acquired from different manufacturers.

Zwiggelaar et al. [52] investigated a combination of statistical modelling and EM algorithm for a texture based approach to mammographic tissue segmentation. The investigation consists of basic grey-level information and spatial correlation information, both combined to achieve texture modelling. The statistical modelling was used for data generalisation and noise removal purposes. Segmentation was derived using an information theoretic approach [132]. The segmented breast parenchyma is in line with breast anatomical structures, and the accuracy was influenced by the size and shape of the local neighbourhood. In a follow-up study, Zwiggelaar et al. [53] incorporated a set permutation-occurrence matrices to encode texture features. The study emphasised that, for texture based mammographic tissue segmentation, it is important to incorporate both grey-level value and spatial correlation information. The use of cooccurrence matrices in texture based segmentation has a major disadvantage, because in principle the dimensionality for the derived texture features is infinite, leading to redundant features and time consuming analysis. Zwiggelaar and Denton [55] addressed this issue by qualitatively selecting a subset of a large set of cooccurrence matrices. A transportation measure [133] was used to determine the difference between cooccurrence matrix based texture features. Segmentation results showed improvements over tissue specific areas when compared to previous studies [52–54]. This indicated that the segmentation performance is directly related to the transportation measure of an ordered set of cooccurrence matrices.

When using the conventional EM based segmentation, the efficiency of the stochastic model depends on the accuracy of estimation of the model's parameter set. Selvan et al. [56] proposed an advanced EM approach to estimate the model parameter set more accurately for mammographic tissue segmentation. Two heuristic search techniques were investigated, particle swarm optimization [134] and evolutionary programming [135]. Experimental results indicated better approximation of a given histogram by a mixture of density functions and reduced computational time when compared to the popular EM algorithm. Visual assessment showed that when compared to the reconstructed image using standard EM parameter estimates, irrelevant image details are largely reduced in the resegmented image where parameters are estimated by the heuristic approach. The usefulness of the optimisation approach depends on accurate estimation of the model parameter set and incorporation of context information.

#### 4.1.3. Statistical Model Building

There are two main types of statistical model building techniques to model breast tissue, those based on texture statistical variation or those which use texture descriptors. These techniques are closely related and can be combined. A summary of representative studies using statistical model building techniques can be found in Table 3.

*Texture Statistical Variation.* Miller and Astley [57] were among the first to perform mammographic tissue segmentation using texture features. Two approaches were investigated, morphological operation based granulometric techniques [136] and Laws texture energy method [137]. In the granulometric based multiresolution method, morphological structuring elements of various sizes were used to cover the range of anatomical structures. Their shape determines the texture characteristic to be measured; in particular, a set of circular structuring elements were used to produce images in which intensity represents texture coarseness, whilst for Laws texture energy based approach, a Bayesian classifier [138] was trained on a leave-one- (image) out basis to produce glandular probabilities for an unseen mammogram. The investigation indicated that breasts with dense-glandular patterns and relatively smooth disc appearances were falsely classified as fatty tissue; the misclassification was linked to poor textural detail as seen on digitised SMF. This may have been due to the quality of digitisation, spatial and grey-level resolution.

Suckling et al. [58] proposed a method to segment mammograms into four classes (i.e., background, pectoral muscle, fibroglandular tissue, and fatty tissue). The first- and second-order grey-level histogram features were calculated and used in multiple linked self-organising neural networks for pixel classification [139]. The developed approach did not use separate data explicitly to train breast tissue models; training was performed over the novel images themselves, at tissue specific areas based on prior knowledge. It was assumed that there is a certain consistency in the patient positioning, and the positioning of anatomical structures is in the same regions. Segmentation results indicated that breast peripheral boundaries were difficult to identify due to X-ray photon penetration which is almost complete over these areas. Visual assessment indicated reasonably good segmentation with a tendency of oversegmentation; however, anatomic structures with variable appearance of the fibroglandular tissue were not identified correctly. The position of the parenchyma alters dramatically across the series of images, but the algorithm does seem to produce consistent parenchymal shape over several images. It was concluded that high density tissue was found to be more prone to misclassification, and the number of neurons used had a direct effect on the performance of the network. This early study indicated that potential image acquisition related information (e.g., the thickness of the compressed beast, the attenuation coefficients of the breast tissue, and breast anatomical models) may be useful for the task but was difficult or unattainable when using digitised SFM.

Petroudi and Brady [61] proposed a statistical modelling approach to mammographic tissue segmentation based on a framework previously developed for mammographic risk classification [140]. The approach focused on texture analysis over a more localised area, and a pixel based classification can be used as a means of segmenting breast parenchymal into different densities. To incorporate both contextual and spatial neighbourhood (structural) information, multivector Gaussian HMRF (Hidden Markov Random Field) [141] and texton (texture primitives) [140] technique were used. The multivector image representation [142] is achieved using a filter bank [143], whilst all the parameters were estimated using the EM algorithm. The key aspect in the study is the use of texton based statistical modelling for rotation invariant mammographic texture. The hypothesis is that pixels from similar breast tissue have similar texture properties, even when there are relatively large anatomical changes due to involution and use of hormone replacement therapy. However, issues still remain with the automatic determination of the appropriate filter bank, the number of textons, and the size of the neighbourhood. The texton selection aspect was investigated in a related study [62], which again used textons and HMRF for mammogram tissue segmentation. Similar segmentation results were achieved when a greedy algorithm was incorporated in the textons learning process to remove similar textons.

Oliver et al. [63] presented a mammographic density segmentation which utilises modelling of a set of patches of either fatty or dense parenchyma and statistical analysis. Two modelling strategies were investigated; one is a Karhunen-Loeve-based model with PCA [144] and the other is a linear-discriminant-based model using LDA [128]. Once the tissue models are learnt, pixel based two-class (i.e., fatty and dense) segmentation was performed using a nearest neighbour classifier. The evaluation was performed using both digitised SFM and FFDM. There were noticeable variations observed in tissue appearance for the two databases; therefore, the breast tissue modelling was independently performed for both databases. The study indicated that the number of patch samples used is vitally important in the training process; it should be large enough to provide sufficient data variation per tissue class but small enough to avoid overfitting of the classifier. It may be difficult to subsample the square or rectangular patches with the precision needed within a mixture of tissue, leading to oversegmentation. A further detailed annotation over patches could be employed to address this issue; however, this procedure can be labour intensive and time consuming. It was also noticed that the algorithm may not be able to correctly identify fatty tissue, some ducts, and linear structures that are much brighter than the fatty breast tissue. This is due to texture modelling variation when using digitised and digital mammograms. Many of the issues related to patch based breast tissue modelling are in line with a number of studies reviewed in Section 4.2.

*Texture Descriptors.* Zwiggelaar and Denton [64] developed a texture classification approach using contrast information, based on the concept that texture can be discriminated from the contrast between key structural elements and their repeating patterns. The developed texture analysis method is related to local binary patterns (LBP) [145] and is similar to SUSAN [146]. Mammographic texture is modelled by estimating local aspects using a set of binary images which can be generated by thresholding using the corresponding grey-level bands. Unlike the LBP and SUSAN approaches, this method does not utilise histogram information to extract texture information, only one model per grey-level band exists, and these models can be compared directly. A hybrid metric based on probability density distributions and transportation estimation was used to classify unseen pixels as a means of segmentation. Results showed a strong correlation between the various texture regions in the mammographic images and the segmented areas. The region boundary effects seem to play a significant role, as most segmented areas were mainly occupied by two classes; this is partly due to unbalanced distribution over the density classes.

Zwiggelaar [66] investigated a breast tissue segmentation methodology based on local grey-level appearance histograms. The mammographic texture modelling incorporated both grey-level and spatial aspects. Variation in local grey-level appearance is represented in histogram format for which the distribution varies with BI-RADS breast density categories. Visual assessment indicated realistic mammographic tissue segmentation but is predominately two-class segmentation associated with BI-RADS II and III, whilst the breast boundary regions are often associated with high risk BI-RADS III and IV. It should be noted that the underlying information within the local window is the same as that being used in texton based approaches [61], where the texture models are formed by cluster centre related histograms. A distinct difference compared to LBP based texture analysis is that the developed methodology uses the full grey-level range instead of reduction to binary patterns, which means the resulting histograms might contain more sparsely populated texture information.

#### 4.1.4. Collective Multiple Measurements

According to the literature, different approaches to breast density estimation can be performed equally well. However, manual/semiautomatic segmentation can remain superior despite some of the inherited limitations (e.g., losing accuracy due to fatigue and time consuming process). It may be that a human observer is able to combine context, morphology, and textural information, whereas many automated methods only focus on one of these characteristics [67]. Therefore, it is conceivable to utilise various principles for mammographic tissue segmentation. A summary of representative studies using collective multiple measurements can be found in Table 4.

Kallenberg et al. [67] developed a breast density segmentation method based on pixel classification, in which different approaches known in the literature to segment breast density are integrated and extended. The features used include, for example, location of clustered high density tissue in relation to the skin line and nipple, dense tissue intensity information, Gaussian derivatives, cooccurrence matrix based Haralick texture features, and global context features calculated from the whole image. A neural network classifier was trained based on segmentations obtained using the Cumulus. The sequential floating forward selection (SFFS) algorithm [147] was applied to select the optimal subset from a large pool of features, which removed irrelevant and redundant features from the data, and a threshold was defined for the classifier output to obtain a dense tissue segmentation. The PD results show a high correlation (*r* = 0.9) between the automatic measurements and the Cumulus results. It was concluded that a combination of segmentation strategies outperforms the application of single segmentation techniques.

Li et al. [68] investigated image-processing software based on ImageJ [148] for automated analysis of mammographic density and penalised regression to construct a measure that mimics the Cumulus. A set of automated thresholding methods were applied to separate the dense breast tissue, and these vary according to the type of pixel intensity information (e.g., histogram shape, clustering, and entropy). A watershed algorithm was employed to subdivide dense tissue areas into smaller objects from which a variety of measurements were obtained for the breast as a whole, as well as for the “objects” of dense tissue. A total of 1008 measurements were obtained as output from ImageJ, but only 772 variables were informative. The feature dimensionality was further reduced to 123 using PCA. To compare breast density as measured by the Cumulus, a large dataset containing cases (having condition/disease) and controls (not having condition/disease) was used for the evaluation. Results indicated that the mammographic density measurement has a correlation equal to *r* = 0.875 which was similar to that reported by Kallenberg et al. [67] (*r* = 0.895) and substantially higher than the work done by Heine et al. [60] (*r* = 0.70). Note that generalisation of the developed method is currently limited to MLO images. So far, this study provided the strongest evidence that mammographic images contain additional information to percentage density which improves the ability to discriminate between breast cancer disease statuses.

#### 4.1.5. Other Methods

There are some methods in the literature which have not been widely adopted, most conducted as proof of concept or feasibility studies. Although no quantitative and qualitative evaluations were performed with respect to mammographic tissue segmentation, some of the ideas are worth noting which may inspire further improvements and investigations. A summary of representative studies can be found in Table 4.

Lao and Huo [149] developed a hierarchical approach to segment breast dense tissue from mammographic images based on unsupervised learning and multiple levels of detail. The method initially segments the breast based on entropy maximum thresholding. Next, the resultant dense and fatty tissue segmentation was used to facilitate FCM membership function initialisation, followed by clustering based segmentation. Finally, the grouped dense tissue was used to generate dense tissue intensity and homogeneity features; a pixel based thresholding technique was used to produce the final dense breast segmentation. Chen et al. [70] developed a mammographic tissue segmentation method using topographic maps of breast regions at multiple intensity levels that represent both topological and geometrical structures of different dense breast tissue. A topographic map is a morphological and multiscale decomposition of an image relying on the connected components of level sets. The topological and geometric structures are represented by a shape tree, from which dense tissue regions are detected by analysing the saliency and independency of the shapes. This is an unsupervised method as it does not require a learning stage or prior knowledge. The analysis is based on components of the topographic map instead of the image pixels, which could significantly reduce the dimensionality of the data to be analysed. Postprocessing is required to compute geometric moments of the level sets in order to remove incorrect dense regions. Visual assessment indicated that the derived segmentation resembled the results obtained using threshold based methods; however, segmentation seems to omit anatomical structures which do not appear as dense as they should be.

### 4.2. 2D Projection Based Approaches Using Parenchymal Pattern

Another group of mammographic tissue segmentation approaches focuses on breast tissue separation beyond different densities (e.g., dense and fatty) and using Tabár's tissue modelling instead to group breast tissue into four classes (i.e., linear, nodular, homogeneous, and radiolucent); see Section 2.2 for details of this scheme.

#### 4.2.1. Statistical Model Building

These methods are all related to statistical model building using texture statistical variation or descriptors. A summary of representative studies with respect to Tabár's tissue modelling can be found in Table 5.

*Texture Statistical Variation.* Muhimmah et al. [71] applied a texton related technique for mammographic tissue segmentation based on Tabár's tissue modelling. A texton selection strategy was incorporated using a combination of visual assessment and minimum spanning tree topological information. The textons for each tissue type (i.e., nodular, linear, homogeneous, and radiolucent) were generated from mammographic patches containing tissue specific samples. In the texton selection process, the (Euclidean) minimum spanning tree was used to indicate a topologically probable correct connectivity, in high dimensional space. Distinct textons (higher discriminative power) tend to be situated towards the outer edges of the tree, whilst common texture, noise, and intensity aspects tend to be modelled by textons in the central part of the tree. Subsequently, a model driven based mammographic tissue segmentation was performed using the selected textons. An alternative texton selection process using texton ranking, outlier detection, and visual assessment can be found in [150]. Both studies partially addressed the texton selection issue discussed in [61] and showed realistic segmentation results.

He et al. [73, 74] investigated a mammographic tissue segmentation based on spatial moments and prior information (e.g., shape related texture features) of mammographic building blocks. A set of geometric moments (spatial moments) of different orders were computed over local textures. They were used as global shape descriptors to encode different spatial characteristics of mammographic texture intensity distribution. Visual assessment indicated that the spatial moments based texture features are able to capture orientation sensitive texture properties, as well as more complicated textural properties, resulting in realistic mammographic tissue segmentation. However, the segmentation has a tendency of oversegmenting linear structures and undersegmenting small areas from nodular and homogeneous regions. Note that a low intensity background (e.g., low or zero grey-level values) can lead to “meaningless moments” [151] which can cause misclassification between high density homogeneous tissue and low density radiolucent tissue. The developed methodology is capable of modelling complex mammographic images and can deal with intraclass variation and noise aspects. It is particularly suitable for modelling Tabár's mammographic building blocks as different breast tissue types have texture patterns related not only to their periodicity but also to shape features.

Clinical observations indicated that breast peripheral areas may not be fully compressed which may cause unexpected intensity and texture variation within these areas. Such breast parenchymal appearance discrepancies may not be desirable for tissue modelling within computer aided mammography. He et al. [76] developed a mammographic image preprocessing (peripheral enhancement) method to improve the image quality before analysis (similar issue was addressed in [152]). Visual assessment indicated significant improvement on segmented anatomical structures and tissue specific areas when using the processed images.

**Figure 6:**
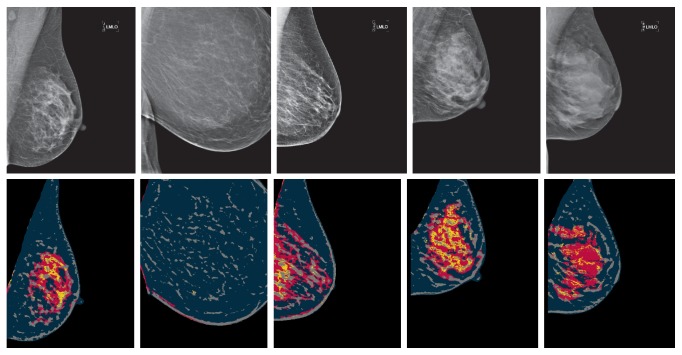
Example mammographic parenchymal segmentation. Images (digital mammograms) from left to right are rated with Tabár I–V, respectively. Nodular, linear structure, homogeneous, and radiolucent areas are colour coded as yellow, grey, red, and navy, respectively.

*Texture Descriptors.* He et al. [77] developed a texture signature [153] based methodology for mammographic tissue segmentation in an attempt to incorporate spatial and geometric texture features. The framework is similar to the moments based approach [74], but for each pixel a texture signature is generated which consists of three distinct subsignatures. It is effectively a stack of three 2D histograms, encoding directional texture features (e.g., intensity, orientation, and elongation variation). Visual assessment indicated good and consistent segmentation results, and it was robust in dealing with the “meaningless moments” issues encountered in [74]. Two aspects were investigated in subsequent studies [75, 78] to further improve the framework. First, a binary model matching pattern based Bayes classifier was investigated in [75]. An idea similar to “fuzzy membership” was used to allow a pixel to be associated with multiple classes at the same time. The process generates a binary pattern of the “fuzzy membership” through model matching and is subsequently used in training the Bayes classifier. Second, a feature and classifier selection technique was proposed in [78]. Results indicated that at the tissue modelling stage, over- and/or undertraining can cause tissue composition fluctuation between nodular and homogeneous tissue, whilst the percentages of radiolucent tissue were less sensitive to the algorithm's parameter configurations. This resulted in inadequate compositions for nodular and homogeneous tissue according to Tabár's parenchymal patterns. Despite the shortcomings in some cases, these improvements helped to produce more realistic segmentation and improved risk classification accuracies. Example mammographic segmentation can be found in Figure 6.

### 4.3. 2D Projection Based Volumetric Approaches

Due to technological limitations, 2D projection area measures are known to be flawed [154, 155] as they do not provide measures of the fibroglandular tissue volume (FVG). With X-ray mammograms, the brightness at any given point in the image is dependent on the thickness of glandular and other dense tissues projected onto that point. The arrangement of glandular tissue within the breast depends on the way in which the breast is compressed. Therefore, measures of the projected dense tissue area vary depending on compression. Substantial efforts have been directed towards volumetric breast density estimation techniques which may allow for improved assessment of breast fibroglandular tissue; this may in turn lead to improved breast cancer risk assessment [156]. Three groups of 2D projection based volumetric approaches can be identified, prior calibration, in-image phantom based calibration, and physical image formation models. A summary of representative studies using 2D projection based volumetric approaches can be found in Table 6.

#### 4.3.1. Prior Calibration

Highnam and Brady [157] developed the Standard Mammogram Form (SMF) to measure glandular tissue composition objectively, with the details in Section 4.3.3. However, only “relative” tissue measurements were obtained unless a careful calibration was performed. Following their study, Kaufhold et al. [158] proposed a calibration approach to glandular tissue composition estimation in digital mammography. The approach was evaluated using breast phantoms of varying thickness and FFDM. From these images, mean signal, noise levels, and computed calibration curves were extracted for quantitatively estimating tissue composition on a pixel-wise basis. In terms of different error sources on the estimates of tissue composition, the initial study concluded that these error sources include compressed breast height estimation error, residual scattered radiation, quantum noise, and beam hardening. Errors in the estimated compressed breast height contributed the most error in tissue composition. Heine et al. [79] proposed a calibration method as an extension to [158] which coupled with first-order histogram statistics as an automated surrogate for breast density assessment from FFDM for the purposes of estimating cancer risk directly. It should be noted that this method requires the use of raw FFDM explicitly. The calibration process produces a normalised effective X-ray attenuation coefficient scale at the pixel level referred to as the percent glandular representation. Mammograms are standardised automatically in this way; the calibration resulted in normalised images with pixel values ranging from 0 to 100, where increasing value represents increased X-ray attenuation. The PD measure is an estimate of the number of pixel values above a fixed idealised X-ray attenuation fraction.

#### 4.3.2. In-Image Reference Phantom Based Calibration

Pawluczyk et al. [159] described a volumetric analysis of the mammographic density from digitised SFM. An initial calibration of the imaging system is required with a tissue-equivalent plastic device. The subsequent correction for exposure factor variations and film processing characteristics is performed through images of an aluminium step-wedge placed adjacent to the breast during imaging. In order to calculate volumetric breast density (VBD), information about the compressed breast thickness and system parameters used for taking the mammogram and information from the calibration device are all taken into account. Results obtained for the known density phantoms showed that VBD can be estimated to be within 5% accuracy of the actual value. However, thickness correction is needed to correct inaccuracy in the compression thickness indicator of the mammography units. Alonzo-Proulx et al. [80] adapted the work of Pawluczyk et al. [159] and proposed a volumetric method which incorporated a more robust algorithm to estimate the thickness of the breast [160, 161] using a prior calibration of the digital image signal versus tissue thickness and composition. The thickness of the compressed breast is estimated using an empirical model that corrects the thickness readout of the mammography system as a function of compression force. The developed VBD measurements are in good agreement with the VBD measured on a dedicated breast CT system [162].

Hufton et al. [163] developed a quantitative method for determining the volume of dense breast tissue from digitised SFM, similar in principle to [159]. This automated technique used a calibrated step-wedge placed alongside the breast during mammography; markers on the compression plate enable accurate measurement of compressed breast thickness and the thickness of dense tissue to be determined at each pixel. Data from the step-wedge and acquired images were then used to measure total breast volume, glandular volume, and the derived percentage of volumetric dense breast tissue. Such a method is insensitive to variations in exposure parameters and film processing. Note that where the breast loses contact with the compression plate (e.g., peripheral area), a rank order filter based on noise estimation is used to find the outer edge and a semicircular profile model is used to determine breast thickness in this region. The original design of the step-wedge (35 mm) was too big to fit alongside larger breasts and the lead lining made it a relatively heavy, unwieldy device that could not be easily attached to the bucky [164]. Identification of step positions in the digital image is required for analysis, but one end of the wedge was overexposed and the other underexposed; therefore finding the ends accurately was challenging.

Shepherd et al. [165] developed a method to measure the percentage fibroglandular tissue (PFV) and FGV using single X-ray absorptiometry (SXA) in order to obtain tissue volumetric measures during image acquisition using a reference phantom. This can generate an accurate thickness profile of the breast at each image position and calculate an image specific calibration of the pixel grey scale values to breast composition. It is a promising method which may be utilised in commercial breast image equipment. Closely related work can be found in [166, 167]. A modified calibration approach for the SXA was proposed in [168]. The method takes into account both geometric and image related factors that impact the calibration of grey-level into absolute tissue composition. Breast density is calculated using the thickness correction factors and recalibration procedures, where all necessary parameters are extracted automatically. Note that it is essential to conduct weekly phantom scanning as part of quality control monitoring, with a specially designed calibration phantom to control thickness and grey-level conversion stability. This weekly maintenance could be a limitation to the SXA approach. To address this, Ourselin et al. [81] developed a method to automatically quantify volumetric breast density without the use of phantoms. It applies a shape and appearance model to digital mammograms and combines volumetric density measures derived by the SXA method to build a statistical model based on image parameters extracted from the image.

#### 4.3.3. Physical Image Formation Models

Highnam and Brady [157] proposed a normalised representation of the breast which was referred to as the Standard Mammogram Form (SMF). Such an approach transforms digitised SFM to an “interesting” tissue representation. The key assumption in the normalisation method is that the X-ray attenuation coefficients of fibroglandular and abnormal tissue are almost equal and are quite different from that of fatty tissue. The technique utilises mammographic information which relates to the physical volume of the breast; the height of nonfatty tissue under compression that corresponds to each pixel is calculated in order to estimate the volume of “interesting” tissue. The volumetric values of “interesting” tissue have a good anatomical correspondence with the expected tissue density of each mammographic risk. A mammogram correction is required for scattered radiation and the dependency of image formation parameters (e.g., tube voltage (kVp), spectrum, and exposure time (mAs)). Nevertheless, such a volumetric breast density estimation is fully automated and is able to compensate for calibration data errors (e.g., mAs, kVp, and spectra) by using image-based calibration [169]. Due to relatively weak control over the image acquisition process, it is difficult to eliminate variability in image characteristics (e.g., contrast and brightness), leading to variation in the mammographic intensity distribution, and increased difficulty in identification of grey-level texture based mammographic patterns [170]. However, closely related studies [171, 172] noted that possible errors can occur in the calculation of tissue height, which influences the overall accuracy of the measures. The main disadvantage of SMF is that a complete and substantial set of calibration data would be needed to generate realistic breast composition measures and yet there are many trials that have retrospectively collected images with no calibration data. The issue of measuring breast composition using retrospective digitised SMF with little or no calibration data available was later addressed in [169]. Such a volumetric technique was criticised as less reliable than 2D threshold based methods [173] and did not provide a stronger predictor of breast cancer risk [174].

van Engeland et al. [175] presented a method for estimation of dense breast tissue volume using FFDM. Such an approach maps the thickness of dense tissue to a pixel, determined using a physical model of image acquisition. Linear attenuation coefficients of breast tissues were derived from empirical data as a function of tube voltage, anode material, filtration, and compressed breast thickness. The developed volumetric breast density estimation can be severely hampered or rendered impossible for contrast enhancement images, because such images violated the assumption made that pixel values are proportional to exposure. Results were significantly worse when applied to images after logarithm transformation (“for presentation/processed” images) than those obtained from the raw images. Over- or underestimation of breast density volume can be seen when the method was applied to thickness corrected images, whilst very dense breasts can cause difficulty in obtaining a reliable calibration location. In contrast to the SMF approach [157], many aspects of the image formation process and parameters related to the acquisition procedure were required, whilst in [175] many calibration parameters can be obtained from the DICOM header. Note that all volumetric techniques from 2D projection data assume two components (i.e., fatty and dense tissue) in the breast; however, more recent volumetric developments (e.g., van Engeland's work) are far less reliant on the image physics, although there is still some sensitivity to compressed breast thickness [176].

#### 4.3.4. Commercial Software

Hartman et al. [82] presented a volumetric breast density technique as an extension to SMF [157]. The software is named Quantra, which significantly improves Highnam and Brady's SMF approach [157]. The performance of SFM based volumetric breast density estimation and breast cancer risk has been reported to be equal to visual assessment methods (e.g., Wolfe and SCC) but poorer than the Cumulus. This may be due to error compensation in the calibration data by finding the breasts fatty peripheral areas which is difficult for very dense breasts, leading to underestimation in volumetric density and undermining breast cancer risk predictions [169].

Highnam et al. [83] built upon the work in [175] and developed software named Volpara that uses relative physics modelling together with additional information derived from the image to substantially reduce dependence on imaging physics data. The volume of dense tissue is found by a ratio of integrated (dense tissue) values over the breast areas, and the volume is calculated by multiplying the area of the breast using the recorded breast thickness. One of the difficulties in volumetric based breast density estimation, as pointed out in [173], is in finding a reference area of the breast which is entirely fat, especially when the breast is very dense.

Both commercial software programmes have been under scrutiny, and reports from different studies are not always in line with each other. For example, a validation study [84] objectively evaluated Volpara breast density assessment on FFDM using measurements obtained from breast MRI. It was concluded that accurate volumetric breast density assessment is feasible in FFDM and has potential to be used in objective breast cancer risk models and screening stratification. Similarly, Wang et al. [177] assessed agreement of three techniques (i.e., SXA, Quantra, and Volpara) with MRI for PFG, absolute FGV, and total breast volume. It was concluded that automated volumetric fibroglandular tissue measures from FFDM were in substantial agreement with MRI and if associated with breast cancer could be used in clinical practice to enhance risk assessment and prevention. However, Jeffreys et al. [178] compared the new volumetric breast density method using Volpara with the Cumulus. Results indicated that there is a very strong correlation between the Cumulus PD and VBD, but there is less relationship between the Cumulus PD and the absolute volume of dense tissue. A recent study [179] has shown disagreement in measured volumetric density when using Quantra and Volpara based technologies. To date there are no reports of breast cancer associations for Volpara or Quantra measures of volumetric breast density techniques. Therefore further validations of these new methods against breast cancer outcomes are needed. It should be noted that volumetric breast density can be measured from 2D projections, but the precise location of the dense tissue cannot be identified. Therefore, to date there are no volumetric segmentation results available. However, Volpara can produce a “density map” which allows dense mammography patterns to be visualised. Promising results [10, 180] emerged recently showing that volumetric density measures have the potential to surpass area based measures in breast cancer risk assessment.

### 4.4. 3D Reconstruction Based Volumetric Approaches

A limited number of studies exist regarding mammographic tissue segmentation using 3D DBT; so far there are few investigations in the literature which lead to developing “true” 3D dense tissue segmentation algorithms for estimating volumetric breast density.

Kontos et al. [181] conducted a feasibility study to distinguish dense and fatty tissue in DBT using texture features (e.g., skewness, coarseness, contrast, energy, homogeneity, and fractal dimension). The selected texture features have been used in previous mammographic tissue segmentation studies (e.g., [44]) and indicated that these features tend to correlate to breast density when computed from DBT images [182]. When computing grey-level texture statistics, the conventional 2D texture descriptors were extended to 3D by considering 3D voxel neighbourhoods rather than 2D pixel neighbourhoods. The study concluded that the fractal dimension was superior in DBT, while contrast was best in 2D projections. It was suggested that for 2D tomographic separation of the breast tissue layers in DBT, the dominant contribution to the grey-level values in the images is the X-ray attenuation at the specific voxel in the breast volume. This indicated that volumetric parenchymal properties such as self-similarity reflected by fractal dimension could be more accurately estimated by the corresponding DBT texture features. Recent improvements on Kontos's method have led to fully automated 3D fibroglandular tissue segmentation and VBD estimation from DBT images, and the study showed strong agreement with existing volumetric techniques based on FFDM and MRI images [183]. The updated algorithm exploits the geometry of the acquisition of DBT sequences as well as the relationship between image intensity and tissue density; 3D segmentation of the fibroglandular tissue is achieved by analysing both 2D projection images and reconstructed DBT slices. Results indicated that VBD estimations were highly correlated for DBT and FFDM (*r* = 0.88), DBT and MRI (*r* = 0.76), and FFDM and MRI (*r* = 0.73). In terms of clinical relevance, such a fully automated quantitative VBD estimation from DBT could result into more accurate measures of the fibroglandular tissue in the breast which may lead to more accurate measure of breast cancer risk.

Shafer et al. [184, 185] conducted a multimodality study which utilised a 3D hidden-Markov model (HMM) based breast tissue modelling technique for DBT segmentation of adipose and glandular tissue. Much work has been done previously using 2D HMRF to estimate density and other features from mammograms [61, 186]. Glandular segmented MRI were used in training a 3D HMM; the model was validated and used to segment DBT breast volumes. The breast density for DBT images is calculated as the ratio of glandular voxels to all voxels in the breast volume. All MR and DBT images were processed to optimise the available range of values for the breast tissue segmentation task. It was also assumed that the possible tissue type of each DBT voxel is either adipose or glandular. The evaluation showed mixed results; some are encouraging and others are dissatisfactory. This indicated that the cross-modality training and testing scheme using HMM needs to be further investigated to refine the process and increase accuracy and reliability for breast DBT segmentation.

## 5. Discussion

**Figure 7:**
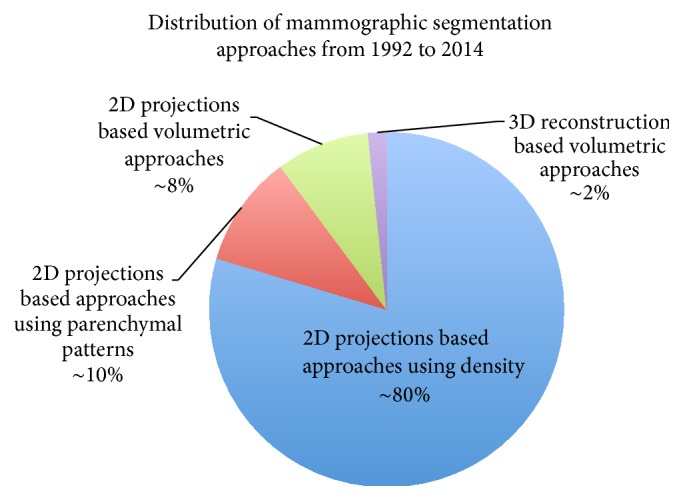
Distribution of various mammographic tissue segmentation approaches from 1992 to 2014.

**Figure 8:**
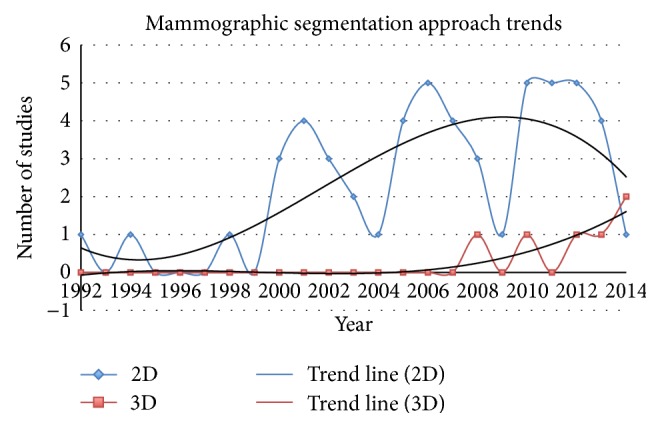
Polynomial based trend lines for both area and volumetric based mammographic density/parenchymal segmentation approaches from 1992 to 2014. Note that the (blue) 2D trend line shows harmonic (e.g., interval peaks) behaviour which may be because a surge of manuscripts was submitted for publication at the same years (e.g., biannual) as the major breast imaging related conferences.

This review covers mammographic tissue segmentation existing in the literature covering the period from 1992 to 2014, focusing on automatic approaches, and excluding manual and semiautomatic approaches. Only a fully automatic approach can deliver consistent, standardised, fully reproducible, and comparable measurements for high-throughput breast screening programmes across sites.  Figure 7 shows the distribution of various mammographic tissue segmentation approaches, and Figure 8 shows the trend lines for area and volumetric based mammographic density/parenchymal segmentation approaches. This indicates a decline in the number of studies using 2D projection and an increase in the number of studies in 3D volumetric segmentation. Without automation and accurate estimation of breast density/parenchymal patterns, mammographic segmentation developed in a research environment is unlikely to become useful in clinical practices for assessing mammographic risk or in clinical trials evaluating the influence of different variables (drugs) on breast tissue [187]. Eng et al. [10] concluded that fully automated methods are valid alternatives to the labour intensive “gold standard” Cumulus for quantifying density. However, the choice of a particular method will depend on the aims and settings, and it is essential that the same density assessments approach is required for a research design or survey in which the same subjects are observed repeatedly over a period of time. Despite various methodology issues related to the developed techniques as reviewed in Section 4, there are several other obstacles for most of the developed approaches to demonstrate that image-based risk prediction can be incorporated in the current non-image-based risk prediction models (e.g., the Gail and the Tyrer-Cuzick models).

### 5.1. Database

There are only two popular publicly available databases (i.e., MIAS and DDSM) containing digitised SFM that have been used in some studies covered in the literature. Only one database (i.e., INBreast [188]) containing FFDM recently became publicly available. Currently there is no tomosynthesis database publicly available, making it difficult to directly compare the robustness of newly developed mammographic tissue segmentation with the existing methods in the literature. Mammographic image appearance varies when using different mammography units and imaging acquisition processes; differences in imaging parameters can affect image brightness, contrast, and textural similarities between very fatty and very dense breasts. The digitisation processes employed for the MIAS, the DDSM, and other private databases are distinctively different, which can also influence mammographic image appearance. A developed mammographic tissue segmentation may work well for one database but not necessarily for other databases; a tuning process may be required to adapt the approach for a different database [189]. As a result, it is difficult to determine the robustness and reliability of a segmentation approach without quantitative and qualitative analysis using mammographic images obtained from different manufactures and perhaps digitised differently. Note that digitised SFM lacks the linearity of the characteristic curve of film, which in combination with the lack of uniformity of thickness of breast tissue during compression can influence the accuracy of the relationship, between mammographic findings of apparently dense breast tissue and the X-ray attenuation of breast tissue [190]. Based on this review and within the scope of automatic mammographic tissue segmentation, ~73% of the studies used digitised SFM, ~25% used FFDM, and <2% used DBT. For the evaluation or validation, about ~35% of the studies used publicly available databases with the rest using private datasets, size varying from a handful of images to in excess of a thousand images.

### 5.2. Imaging Modality

There are doubts about the relationship between mammographically determined breast density/parenchymal patterns and breast cancer risk [155]. One of the inherent limitations for area based measurements is that they do not take thickness of dense tissue into account. It is biologically more plausible that breast cancer risk is related to the volume of dense tissue in the breast rather than to its projection. Conventional 2D mammography is affected by tissue superimposition, whilst volumetric analysis of breast tissue through emerging tomographic breast imaging modalities has been suggested as necessary to advance breast cancer risk modelling [155]. It should be noted that all the mammographic risk/density schemes as discussed in Section 2 were established based on digitised SFM. Although some image-based models like the BI-RADS scheme are reviewed in a clinical environment, there are no updates in the literature to indicate that when using FFDM or DBT, the relationship between breast density/parenchymal patterns and breast cancer risk is the same as that established for SFM. To date, all the studies presume the relationship between breast density/parenchymal patterns of different modalities and breast cancer risk is the same, with no clinical evidence or proof. Previously unknown relationships between breast density/parenchymal patterns and breast cancer risk may be discovered by using more advanced breast imaging techniques and modalities, especially when FFDM is likely to replace standard SFM and DBT is fundamentally different to SFM and FFDM.

### 5.3. Raw or Processed FFDM

Physical image formation model based volumetric approaches use known X-ray attenuation of breast tissue to calculate tissue composition at a given pixel. Successful application of these methods to digitised SFM was initially met with difficulty; however, with the introduction of FFDM, the development of robust methods and commercial products (i.e., Quantra and Volpara) became possible. How the raw images are processed is largely unknown to research institutions as they are manufactured at the proprietors discretion. Experimental results indicated that such a processing is likely to distort the relationship between the image signal and X-ray transmission, and it could interfere with the ability to derive density information from the images [86]. Visually speaking, radiologists have observed that the processed mammograms appear to be less dense than when imaged with film mammography (e.g., see Figures 5 and 6). Therefore, using thresholding or physical image formation model based approaches to measure breast density on a processed FFDM is likely to be problematic, especially if such measurements are to be compared to those obtained from SFM [86].

### 5.4. Study Population Matters

During evaluations, many studies only used a small amount of data from the general populations of western developed countries, and few studies subgrouped data based on different criteria. Although it is well established that density measurements are associated with breast cancer risk, still several aspects are questioned [87]. For example, hormone therapy is known to increase breast density; certain regimes are known to relate to breast cancer risk [191], but the degree to which density and risk are related during hormone therapy is not known [192, 193]. Several studies [96, 97, 194, 195] on the assessment of breast cancer risk in postmenopausal women showed no clear correlation between the disease and tissue density, and the magnitude of the association varied. Therefore studies involving all ranges and subgroups of the populations based on age, body mass, ethnic background, and so forth could be beneficial in providing additional evidence and linking breast density/parenchymal patterns with other breast cancer risk factors established in non-image-based risk prediction models.

### 5.5. Segmentation Schemes

The vast majority of mammographic tissue segmentation approaches are focused on the well-established density as biomarker. However, some of the studies use BI-RADS density categories and others may use the SCC or Wolfe scheme. The relation between breast cancer risk and density is at best hypothesised [87], and the optimal approach to derive information from mammograms relating to risk of breast cancer is still argued [196, 197]. Various classification/categorisation mappings between different schemes have been studied [24, 90, 94], which is beneficial for experimental comparison when using different image-based breast cancer risk prediction models. Recent studies have indicated that not just density but also heterogeneity in mammograms can be associated with risk [196, 198, 199]. This suggested that mammographic tissue segmentation based on Tabár's parenchymal patterns (i.e., mammographic building blocks) could be more useful than density based approaches.

### 5.6. Evaluation

Clinically obtained “ground truth” is often used in algorithm evaluation and validation. Many studies have shown issues of large interobserver variations in breast tissue annotated by radiologists with different mammogram reading experiences. This makes area overlapping based evaluation unreliable. Such variations also exist for mammographic risk assessment by radiologists, as manual mammographic risk assessment is intrinsically subjective, and there is substantial inter- and intraobserver variability [119, 200]. This subjectiveness includes visual assessment. Therefore, inconsistent results could be obtained if a different group of radiologists is used to obtain “ground truth” for evaluation or validation, even when the methodology remains the same. There is no easy solution to overcome this, but using consensus data is probably by far the best way to deal with this issue.

## 6. Conclusions

A comprehensive review on automatic breast density and parenchymal segmentation is presented, suggesting that there are many issues related to the developed techniques, evaluations, and practicalities that hinder the progress of bridging translational research and clinical utilisation. Various fully automatic mammographic tissue segmentation approaches have been developed or are currently under investigation to overcome subjective estimation of tissue composition, reduce inter- and intraobserver variability, and eliminate ambiguous outcomes. The likely transition to use 3D technology with tomosynthesis will provide a platform for work on breast tissue segmentation with 3D rendering to facilitate new areas of work such as 3D image guided biopsy or image guided surgical excision. Mammographic tissue segmentation has a strong impact on breast cancer risk estimation; however, it has limited influence in clinical decision making and has not yet been included in any established risk prediction model. It may be ideal to incorporate image-based risk factors in non-image-based risk prediction models, so that automatically measured breast tissue composition can be used for risk stratification to adapt screening interval to risk. Further investigations are needed in order to develop a robust, reliable, and fully automatic mammographic segmentation which can help to provide prognostic value in distinguishing which women with certain mammographic appearance are likely to develop breast cancer. Only precise identification of women at risk can lead to a successful prevention and/or treatment and a reduction in the incidence of and mortality from breast cancer.

## Figures and Tables

**Table 1:** Summary of representative studies using thresholding based methods for mammographic tissue segmentation. *r*/*R* denotes correlation coefficient. Note that (1) largely identical studies are excluded in the list and (2) in case of multiple results, only the best reported results are listed.

Study	Year	Number of density categories	Modalities	Number of views	Number of images	Segmentation evaluation	Risk/density estimation accuracy
Global thresholding							
Matsubara et al. [25]	2000	Fatty, mammary gland diffuseness, nonuniform high density, and high density	Digitised SFM	MLO	148	Visually assessed	90% (ratios of the four densities)
Saha et al. [26]	2001	Fatty and dense	Digitised SFM	MLO and CC	174	Visually assessed (acceptable)	N/A
Sivaramakrishna et al. [27]	2001	Fatty and dense	Digitised SFM	CC	32	Visually assessed	Spearman's *r* = [0.92, 0.95] (automatic-manual)
Olsén and Mukhdoomi [28]	2007	Fatty and glandular	Digitised SFM	MLO and CC	160 (MIAS + DDSM)	Visually assessed	N/A
Tzikopoulos et al. [29]	2011	Fatty and fibroglandular	Digitised SFM	MLO and CC	322 (mini-MIAS)	Visually assessed	86% (fatty, fatty-glandular, and dense-glandular)
Lu et al. [30]	2007	Fatty and dense	FFDM (raw)	CC	172	Visually assessed	intraclass *r* = 0.94 (BI-RADS)
Ferrari et al. [31]	2000	Fatty and fibroglandular	Digitised SFM	MLO	66 (mini-MIAS)	Visually assessed (84% successful)	N/A
Ferrari et al. [32]	2004	Uncompressed-fatty, fatty, nonuniform-dense, and high-dense	Digitised SFM	MLO	84 (mini-MIAS)	Visually assessed (81% excellent or good)	N/A
El- Zaart [33]	2010	Fatty and fibroglandular	Digitised SFM	MLO	N/A	Visually assessed	N/A

Adaptive/dynamic thresholding							
Zhou et al. [34]	2001	Fatty and dense	Digitised SFM	MLO and CC	260	Visually assessed (94% correct); *r* (CC, MLO) = 0.94, 0.91 (automatic-manual)	42% (BI-RADS)
Neyhart et al. [35]	2002	Radiolucent and radiodense	Digitised SFM	MLO and CC	N/A	Visually assessed	N/A
Kim et al. [36]	2010	Fatty and dense	FFDM	MLO and CC	80	Visually assessed; *r* = 0.99 (automated-manual)	N/A
Nickson et al. [37]	2013	Dense and fatty	Digitised SFM	CC	5919 women	Visually assessed; 41% “perfect” agreement (Cumulus-AutoDensity)	Pairwise *r* = 0.63 (Cumulus-AutoDensity)

**Table 2:** Summary of representative studies using clustering based methods for mammographic tissue segmentation. *r*/*R* denotes correlation coefficient. Note that (1) largely identical studies are excluded in the list and (2) in case of multiple results, only the best reported results are listed.

Study	Year	Number of density categories	Modalities	Number of views	Number of images	Segmentation evaluation	Risk/density estimation accuracy
General clustering							
Oliver et al. [38]	2005	Fatty and dense	Digitised SFM	MLO	180 (MIAS)	N/A	73% (fatty, glandular, and dense)
Strange et al. [39]	2013	Dense and fatty	FFDM	CC	12	Visually assessed	N/A
Marias et al. [40]	2005	Dense, semidense, and fatty	Digitised SFM	MLO	146	Visually assessed	65% (BI-RADS I and II) 86% (BI-RADS III and IV)
Oliver et al. [41]	2005	Dense and fatty	Digitised SFM	MLO	300 (DDSM)	N/A	47% (BI-RADS)
Oliver et al. [42]	2005	Dense and fatty	Digitised SFM	MLO	320 (MIAS) and 300 (DDSM)	N/A	48% MIAS, 47% DDSM (BI-RADS)
Oliver et al. [43]	2007	Dense and fatty	Digitised SFM	MLO	322 (MIAS)	N/A	78% (BI-RADS)
Oliver et al. [44]	2008	Dense and fatty	Digitised SFM	MLO and CC	322 (MIAS) and 831 (DDSM)	Visually assessed	77% MIAS, 86% DDSM (BI-RADS)
Oliver et al. [45]	2006	Dense and fatty	Digitised SFM	MLO	322 (MIAS)	Visually assessed	82% (BI-RADS)
Torrent et al. [46]	2008	Dense and fatty	FFDM	MLO and CC	300	Visually assessed	82% MLO, 75% CC (BI-RADS)
Tortajada et al. [47]	2012	Dense and fatty	Digitised SFM and FFDM	MLO and CC	322 (MIAS), 831 (DDSM), and 236 (digital DB)	Visually assessed	86% MIAS, 77% DDSM, and 92% digital DB (BI-RADS)

Adaptive/modified fuzzy *C*-means							
Chen and Zwiggelaar [48]	2010	4 densities	Digitised SFM	MLO	N/A	Visually assessed	N/A
Keller et al. [49]	2011	2–9 densities	FFDM (processed)	MLO	160	Visually assessed; *r* = 0.75 (automatic-manual)	N/A
Keller et al. [50]	2012	2–13 densities	FFDM (processed and raw)	MLO	160	Visually assessed; *r* (raw, processed) = 0.82, 0.85 (automatic-manual)	N/A

Expectation-maximisation							
Aylward et al. [51]	1998	Dense, fatty, and uncompressed-fatty	Digitised SFM	MLO and CC	70	Visually assessed	N/A
Zwiggelaar et al. [52]	2002	4–6 densities	Digitised SFM	MLO	263 (MIAS)	Visually assessed	67% (SCC)
Zwiggelaar et al. [53]	2003	4–6 densities	Digitised SFM	MLO	263 (MIAS)	Visually assessed	86% (SCC)
Zwiggelaar et al. [54]	2003	4–6 densities	Digitised SFM	MLO	263 (MIAS)	N/A	86% (BI-RADS)
Zwiggelaar and Denton [55]	2004	4 densities	Digitised SFM	MLO	36 (MIAS)	Visually assessed	75% (4 densities: 0%–10%, 11%–25%, 26%–50%, and 51%–75%)
Selvan et al. [56]	2006	5/8 densities	Digitised SFM	MLO	112 (mini-MIAS)	Visually assessed (92% good/excellent)	N/A

**Table 3:** Summary of representative studies using statistical model building based methods for mammographic tissue segmentation. *r*/*R* denotes correlation coefficient. Note that (1) largely identical studies are excluded in the list and (2) in case of multiple results, only the best reported results are listed.

Study	Year	Number of density categories	Modalities	Number of views	Number of images	Segmentation evaluation	Risk/density estimation accuracy
Texture statistical variation							
Miller and Astley [57]	1992	Fatty and dense	Digitised SFM	MLO	40	Visually assessed; 80% overlapping areas matched	N/A
Suckling et al. [58]	1995	Fatty and fibroglandular	Digitised SFM	MLO	30 (15 pairs)	Visually assessed; 69% ± 12% overlapping areas matched	N/A
Heine and Velthuizen [59]	2000	Fatty and dense	Digitised SFM	MLO and CC	50	Visually assessed	N/A
Heine et al. [60]	2008	Dense and fatty	Digitised SFM	MLO and CC	369 cases and 712 controls	Visually assessed	Pearson *R* (CC) = 0.70 (automatic-Cumulus) Spearman's *r* (CC) = 0.49 (automatic-BI-RADS)
Petroudi and Brady [61]	2006	Dense, fatty, and breast edge	Digitised SFM	MLO and CC	32	Visually assessed (88% very satisfactory); ~94.4% overlapping areas matched	N/A
Gong et al. [62]	2006	Dense, dense with structures, fatty, and fatty breast edge	Digitised SFM	MLO and CC	43	Visually assessed; ~87.9% overlapping areas matched based on 15 images	~87.9% (Wolfe)
Oliver et al. [63]	2010	Dense and fatty	Digitised SFM and FFDM	MLO and CC	322 (MIAS) and 250 (the Trueta DB)	Visually assessed; accuracy 0.916 ± 0.038, area overlap 0.900 ± 0.122, and Dice coefficient 0.943 ± 0.077 (60 MLO from the Trueta DB)	N/A

Texture descriptors							
Zwiggelaar and Denton [64]	2006	4 densities	Digitised SFM	MLO	60	Visually assessed	72% (Wolfe)
Adel et al. [65]	2007	Fibroglandular, fatty, and uncompressed and fatty	Digitised SFM	MLO and CC	50 (mini-MIAS)	Visually assessed (68% good); >60% agreement with manual segmentation	N/A
Zwiggelaar [66]	2010	4 densities	Digitised SFM	MLO	322 (MIAS)	Visually assessed	64% (BI-RADS)

**Table 4:** Summary of representative studies using other less popular methods (e.g., collective multiple measurements) for mammographic tissue segmentation. *r*/*R* denotes correlation coefficient; AUC denotes area under ROC curve. Note that (1) largely identical studies are excluded in the list and (2) in case of multiple results, only the best reported results are listed.

Study	Year	Number of density categories	Modalities	Number of views	Number of images	Segmentation evaluation	Risk/density estimation accuracy
Collective multiple measurements							
Kallenberg et al. [67]	2011	Dense and fatty	Digitised SFM	MLO	1300	Visually assessed; Pearson *R* (percent density, dense area) = 0.911, 0.895 (automatic-Cumulus)	N/A
Li et al. [68]	2012	Dense and fatty	Digitised SFM	MLO	765 cases and 747 controls	Visually assessed; *r* = 0.884 (automatic-Cumulus); AUC = 0.589 (four densities: <5%, 25%–50%, 50%–75%, and >75%)	N/A

Other methods							
Chen et al. [69]	2012	Dense and fatty	Digitised SFM	MLO	321 (MIAS)	Visually assessed	70% (BI-RADS)
Chen et al. [70]	2013	Dense and fatty	Digitised SFM	MLO and CC	321 (MIAS) and 831 (DDSM)	Visually assessed	76% MIAS, 81% DDSM (BI-RADS)

**Table 5:** Summary of representative studies using statistical model building methods for mammographic tissue segmentation. Note that (1) this list consists of studies mainly related to Tabár tissue modelling and (2) in case of multiple results, only the best reported results are listed.

Study	Year	Number of mammographic building blocks	Modalities	Number of views	Number of images	Segmentation evaluation	Risk/density estimation accuracy
Texture statistical variation							
Muhimmah et al. [71]	2007	Linear, nodular, homogeneous, and radiolucent	Digitised SFM	MLO	320 (MIAS)	Visually assessed	N/A
He et al. [72]	2008	Linear, nodular, homogeneous, and radiolucent	Digitised SFM	MLO	320 (MIAS)	Visually assessed	N/A
He et al. [73]	2009	Linear, nodular, homogeneous, and radiolucent	Digitised SFM	MLO	320 (MIAS)	Visually assessed (65% good/very good) Tabár (38% good/very good) BI-RADS	53% (Tabár), 70% (BI-RADS)
He et al. [74]	2011	Linear, nodular, homogeneous, and radiolucent	Digitised SFM	MLO	320 (MIAS)	Visually assessed	53% (Tabár)
He et al. [75]	2012	Linear, nodular, homogeneous, and radiolucent	Digitised SFM	MLO	320 (MIAS)	Visually assessed	85% (Tabár), 78% (BI-RADS)
He et al. [76]	2014	Nodular, homogeneous, and radiolucent	FFDM	MLO and CC	360	Visually assessed	79% (Tabár), 80% (BI-RADS)

Texture descriptors							
He et al. [77]	2010	Linear, nodular, homogeneous, and radiolucent	Digitised SFM	MLO	320 (MIAS)	Visually assessed	78% (Tabár), 75% (BI-RADS)
He and Zwiggelaar [78]	2013	Nodular, homogeneous, and radiolucent	FFDM	MLO and CC	360	Visually assessed	N/A

**Table 6:** Summary of representative studies using 2D projection based volumetric methods for mammographic tissue segmentation. *r*/*R* denotes correlation coefficient. Note that in case of multiple results, only the best reported results are listed.

Study	Year	Number of density categories	Modalities	Number of views	Number of images	Segmentation evaluation	Risk/density estimation accuracy
Prior calibration							
Heine et al. [79]	2011	Dense and fatty	FFDM (raw)	CC	106 cases and 106 controls	Visually assessed; linear *R* = 0.78 (automatic-Cumulus)	Risk estimates associated with the lowest to highest quartiles, odds ratios: 1.0, 3.4, 3.6, and 5.6

In-image reference phantom based calibration							
Alonzo-Proulx et al. [80]	2012	Dense and fatty	FFDM	CC	55087	Pearson *r* (left, right volumetric density-breast volume) = 0.92, 0.91	N/A
Ourselin et al. [81]	2014	Dense and fatty	FFDM	CC	480	Percent breast fibroglandular volume, *r* ^2^ = 0.8 (automatic-predicted)	N/A

Physical image formation model							
Hartman et al. [82]	2008	Dense and fatty	FFDM and MRI	MLO and CC	550 (275 pairs) and 88 MRI	Breast density volumes, Pearson (left-right breast, Quantra-MRI) *r* = 0.923, 0.937	N/A
Highnam et al. [83]	2010	Dense and fatty	FFDM and MRI	MLO and CC	2217 and MRI from 26 younger women	Breast density volumes, Pearson *r* (left-right breast, CC-MLO view, Volpara-MRI) = 0.923, 0.915, 0.94	N/A
Gubern-Mérida et al. [84]	2014	Dense and fatty	FFDM and MRI	MLO and CC	680 and 168 MRI	Pearson *r* (volumetric breast density Volpara-MRI, fibroglandular tissue volume Volpara-MRI) *r* = 0.91, 0.84	Density grade, *r* = 0.40 (Volpara-BI-RADS)
